# Supplementary material for: Effect of Protein Content on Heat Stability of Reconstituted Milk Protein Concentrate under Controlled Shearing
Source: Foods. 2024 Jan 14;13(2):263. doi: 10.3390/foods13020263 (PMC10815205; doi:10.3390/foods13020263)

Supplementary Material

Effect of Protein Content on Heat Stability of Reconstituted Milk Protein Concentrate under Controlled Shearing

Anushka Mediwaththe, Thom Huppertz, Jayani Chandrapala, Todor Vasiljevic

Content

Figure S1. Score plots of principal component analysis of FTIR spectra obtained within the Amide I region from 4% RMPC dispersions sheared at 100 s^{-1}, 1000 s^{-1} or 1500 s^{-1} during heating at 90 °C for 5min. (A) or 121°C for 2.6 min (B) and 8% MPC suspensions sheared at 100 s^{-1}, 1000 s^{-1} or 1500 s^{-1} during heating at 90 °C for 5min. (C) or 121°C for 2.6 min (D).

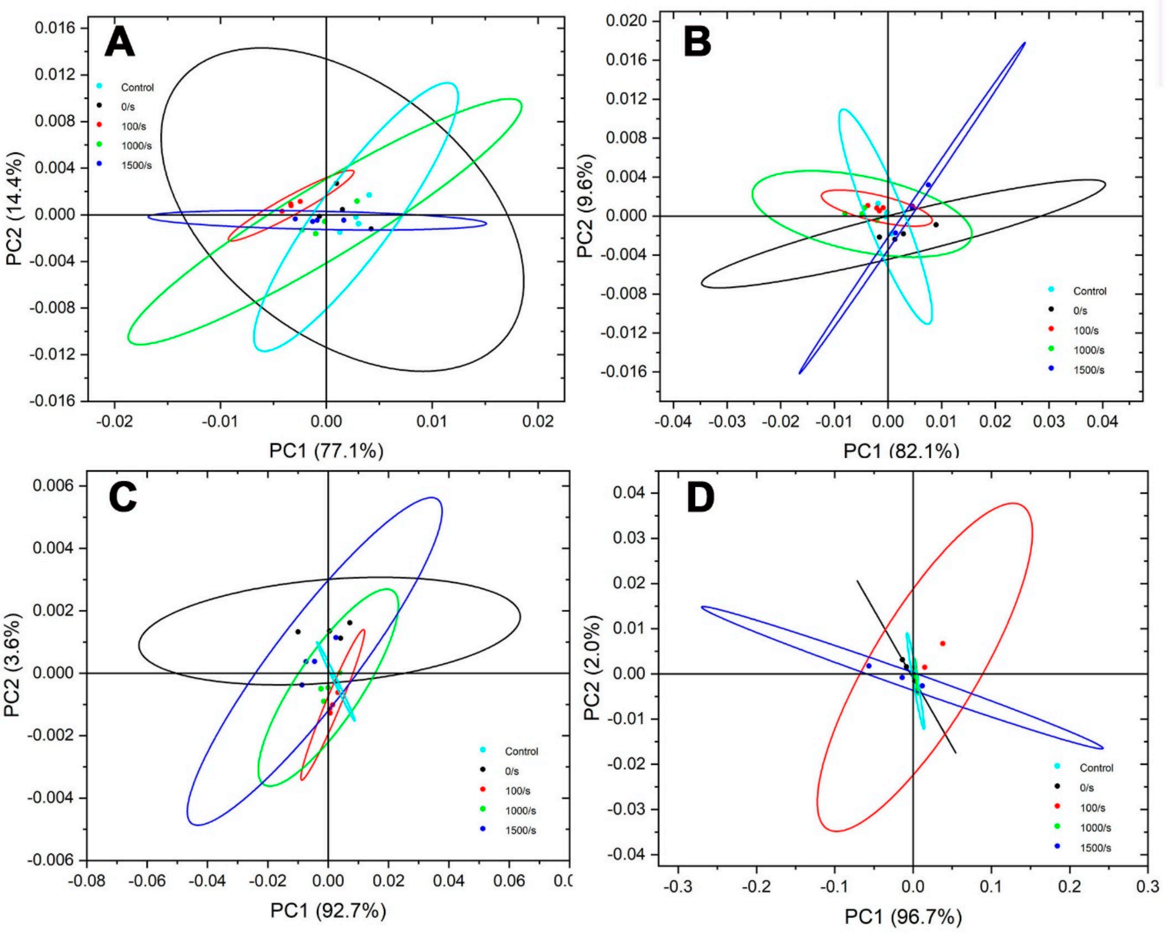

Supplement: Supplementary file 1 [file foods-13-00263-s001.zip › foods-2826004-supplementary.pdf]
